# Hyperthermia-targeted rectal delivery of thermosensitive liposomal doxorubicin via intra-arterial and intravenous administration

**DOI:** 10.3389/fphar.2026.1760944

**Published:** 2026-02-18

**Authors:** Andrew S. Mikhail, Ivane Bakhutashvili, William F. Pritchard, Dieter Haemmerich, Rochel Hecht, Reza Seifabadi, Matthew F. Starost, Rachel Ashe, Keith T. Schmidt, William D. Figg, Bradford J. Wood, John W. Karanian, Michal Mauda-Havakuk

**Affiliations:** 1 Center for Interventional Oncology, Radiology and Imaging Sciences, Clinical Center, National Institutes of Health, Bethesda, MD, United States; 2 Department of Pediatrics, Medical University of South Carolina, Charleston, SC, United States; 3 Division of Veterinary Resources, National Institutes of Health, Bethesda, MD, United States; 4 Clinical Pharmacology Program, National Cancer Institute, National Institutes of Health, Bethesda, MD, United States

**Keywords:** colorectal neoplasms, doxorubicin, hyperthermia, intra-arterial infusion, thermosensitive liposomes, nanomedicine, rectal cancer, pharmacokinetics

## Abstract

**Introduction:**

Lyso-thermosensitive liposomal doxorubicin (LTLD) is a thermosensitive nanomedicine designed to release doxorubicin rapidly at mild hyperthermic temperatures. Unlike systemic doxorubicin, which is limited by cardiotoxicity and poor tumor penetration, LTLD enables targeted drug delivery enhanced by localized hyperthermia through heat-triggered release. While LTLD has demonstrated improved drug delivery with tumor-localized hyperthermia, comparative analyses of intravenous (IV) versus intra-arterial (IA) delivery routes for rectal targeting remain unexplored. This study evaluates doxorubicin pharmacokinetics and rectal tissue accumulation following LTLD administration via IV or IA routes, with or without localized rectal hyperthermia in swine, to identify the optimal delivery strategy for maximizing rectal drug concentrations while minimizing systemic exposure.

**Methods:**

Eight healthy swine were assigned to four groups: IV LTLD with or without rectal hyperthermia, IA free doxorubicin with hyperthermia, or IA LTLD with hyperthermia. Animals received 30-min drug infusions (0.7 mg/kg) via the jugular vein or by bilateral selective catheterization of the internal iliac arteries. Serial blood samples were collected for 1 hour, followed by post-mortem tissue collection from the rectal wall, heart, and perirectal fat. A custom rectal heating device produced homogeneous localized hyperthermia.

**Results:**

IV and IA LTLD combined with localized hyperthermia markedly increased doxorubicin accumulation (µg/g) in rectal tissue (7.45 ± 6.18, 8.41 ± 5.15, respectively) compared with normothermic IV LTLD (0.49 ± 0.16) or hyperthermic IA free-drug controls (0.67 ± 0.46). Plasma AUC_0–60min_ (µg/mL·min) was lowest with IA administration of free drug (12.7 ± 8.36) compared to IV LTLD with and without hyperthermia (424 ± 85.6, 544 ± 148, respectively) and IA LTLD with hyperthermia (305 ± 221). Doxorubicin concentrations in the heart did not differ among treatment groups. Fluorescence microscopy confirmed enhanced doxorubicin distribution within the rectal wall when LTLD was delivered via either route and combined with rectal hyperthermia.

**Conclusion:**

Intravenous and intra-arterial LTLD combined with localized rectal hyperthermia produced similar increases in rectal doxorubicin concentrations in a swine model. These findings support the feasibility of integrating thermosensitive liposomal drug delivery with localized rectal hyperthermia and intra-arterial catheter-based delivery.

## 1 Introduction

Colorectal cancer is the third most common cancer in the United States. Although the overall incidence has declined significantly for several decades (Siegel et al., 2024), the annual percent increase in incidence among adults under 50 years of age was 4% between 2012 and 2022 (SEER*Explorer: an interactive website for SEER cancer statistics, 2025). The rising trend for this age group is primarily specific to tumors in the distal colon and rectum (Siegel et al., 2017). Improper bowel function following colon cancer surgery is common and can be persistent (Verkuijl et al., 2021), with some patients requiring permanent colostomy.

Neoadjuvant and adjuvant therapies for stage II or stage III rectal cancer often include locoregional treatment due to the relatively high risk of locoregional recurrence. This risk is attributed to the rectum’s proximity to pelvic structures and organs, the absence of a serosal layer, and technical challenges in achieving wide surgical margins during resection (Benson et al., 2018). Although neoadjuvant radiation therapy reduces local recurrence rates, it is associated with substantial side effects such as radiation-induced injury and hematologic toxicity (Rahbari et al., 2013).

Randomized trials have evaluated the effectiveness of chemoradiotherapy, which combines concurrent chemotherapy with radiation therapy, administered either preoperatively after clinical evaluation and staging or postoperatively after pathologic staging (Gérard et al., 2006; Sauer et al., 2004). The addition of chemotherapy to radiation therapy may provide local radiosensitization and systemic disease control by eradicating micrometastases. Preoperative chemoradiotherapy has the potential to increase rates of pathologic complete response and sphincter preservation. In a large clinical trial, neoadjuvant chemoradiotherapy resulting in complete or intermediate tumor regression was associated with improved long-term outcomes in rectal carcinoma, independent of clinicopathologic parameters (Fokas et al., 2014). However, while 50%–60% of patients are downstaged following neoadjuvant therapy, only 20% of patients achieve a pathologic complete response (Collette et al., 2007; Lim et al., 2019). A recent groundbreaking randomized trial demonstrated that, for patients with a clinical complete response to neoadjuvant chemoradiotherapy, an organ-preserving strategy without surgical resection was effective in treating rectal cancer (Thompson et al., 2024).

Lyso-thermosensitive liposomal doxorubicin (LTLD) consists of doxorubicin encapsulated within a thermosensitive lipid bilayer that rapidly releases its payload at mild hyperthermic temperatures (39 °C–42 °C) (Needham et al., 2000; Borys and Dewhirst, 2021; Burke et al., 2018; Haemmerich et al., 2023). LTLD has been evaluated in Phase III clinical trials in combination with radiofrequency ablation of hepatocellular carcinoma (Borys and Dewhirst, 2021; T et al., 2018), and in a Phase I trial in recurrent breast cancer (Zagar et al., 2014). Preclinical studies in swine have demonstrated enhanced doxorubicin delivery to the urinary bladder using thermosensitive liposomes and localized hyperthermia, resulting in superior drug accumulation in the bladder wall compared to intravenous (IV) or intravesical administration of free doxorubicin (Mikhail et al., 2017; van Valenberg et al., 2021).

Here, we demonstrate the use of a custom rectal heating device to provide localized hyperthermia to the rectum in swine. We hypothesized that intra-arterial (IA) infusion of LTLD into the arteries supplying the rectum, combined with local mild hyperthermia, would enhance doxorubicin delivery to the rectal wall compared to arterial infusion of free doxorubicin. We also evaluated differences in doxorubicin deposition in the rectum between IV and IA LTLD infusions with rectal hyperthermia.

## 2 Materials and methods

### 2.1 Rectal heating device

**FIGURE 1:**
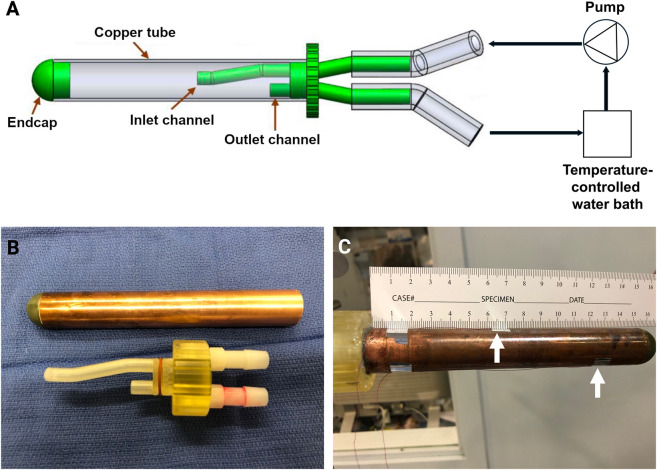
Rectal heating device design and fabrication. **(A)** CAD schematic; **(B)** copper tube with endcap and flow system with inlet and outlet channels; and **(C)** assembled rectal heating device with two thermocouples on the outer surface of the copper tube (arrows).

The prototype rectal heating device was designed and constructed using a hollow, heat-conductive copper cylinder (15 cm length, 2.23 cm outer diameter (OD), 2.0 cm inner diameter (ID)) (Figure 1A). One end of the cylinder was fitted with a 3D-printed endcap and flow channel assembly (Figure 1B). The flow channel assembly included discrete inlet and outlet water channels, as well as a groove containing a rubber O-ring to ensure a watertight seal with the copper tube. The inlet channel was designed to eject water from the cross-sectional center toward the closed end of the cylinder. The endcap had a hollow, rounded region protruding 1 cm from the end of the cylinder.

A 50 L/min submersible pump (AE-172, AquaEuroUSA, Los Angeles, United States) was connected to the inlet channel with rubber tubing (2 m length, 1.7 cm OD, 0.95 cm ID). The outlet channel was connected to an external, variable-temperature water bath and the flow channel assembly was affixed to the copper tube using epoxy adhesive.

### 2.2 Qualitative characterization of rectal heating in a thermochromic phantom

To assess heat distribution, we performed a benchtop test using a tissue-mimicking thermochromic gel phantom that changes color from tan to magenta when heated from 40 °C–70 °C. Synthesis of this polyacrylamide-based gel phantom was performed as previously described (Negussie et al., 2016; Mikhail et al., 2016; Eranki et al., 2019). The rectal heating device was wrapped in polyolefin film and fixed at the center of a 1 L glass beaker filled with the gel phantom precursor solution. After solidifying overnight, the phantom was equilibrated in a 37 °C insulated water bath to match normal physiologic temperature. A separate water bath was heated to 65 °C and the water pump was set to its maximum flow rate (50 L/min) to circulate water through the rectal heating device. This temperature was selected to ensure visible color change in the phantom, enabling assessment of spatial heating homogeneity. After 1 h of water circulation, the rectal heating device was removed, and the phantoms were cut longitudinally or axially and photographed.

### 2.3 Computational modelling of tissue heating and drug delivery

Rectal tissue heating and doxorubicin delivery were simulated using computer models as previously described, with modifications (Mikhail et al., 2017; Gasselhuber et al., 2012). The rectum was modeled as cylinder of 25 mm inner diameter with 3 mm thickness. The model included surrounding tissue, assuming axisymmetric geometry. In addition, the model included systemic plasma and systemic tissue compartments. Briefly, the heat transfer model assumed surface tissue heating from circulating water at 44 °C, heat conduction into the wall of the rectum, and cooling via blood perfusion. The drug delivery model considered temperature-dependent intravascular release of doxorubicin, extravasation of free doxorubicin, and cellular doxorubicin uptake (Mikhail et al., 2017; Gasselhuber et al., 2012). Plasma pharmacokinetics of LTLD were considered based on clinical data from a Phase I trial (Wood et al., 2012). Since no cellular uptake parameters for colon cells was available in the literature, we used prior parameters derived from *in vitro* studies in hepatocytes (Rossmann et al., 2017). IV and IA infusion of drug were simulated by either administering LTLD to the systemic plasma compartment (IV), or to the capillary bed of the rectum (IA) for 30 min. Heating was assumed to continue for a total of 60 min after the start of the infusion, and the heating device was assumed to have been pre-heated as in the animal studies.

### 2.4 Animal model

All animal procedures were approved by the Animal Care and Use Committee of the NIH Clinical Center and conducted in accordance with applicable federal regulations. Eight female Yorkshire swine (16–18 weeks old, 53–63 kg; Oak Hill Genetics, Ewing, IL) were studied. Due to a known anaphylactoid reaction to liposomal formulations, all swine received premedication as previously described (Mikhail et al., 2017). Dexamethasone (0.12 mg/kg, IM) was administered twice daily for 48 h prior to the day of the study. On the day of study, dexamethasone (0.12 mg/kg, IM), famotidine (0.5 mg/kg, IM) and diphenhydramine (2 mg/kg, IM) were given 1.5–3 h before infusion of study formulations. Finally, meloxicam (0.3 mg/kg, IV) was given 10 min before infusion of study formulations. Anesthetic induction was achieved with IV propofol (1 mg/kg) following sedation with IM ketamine (25 mg/kg), midazolam (0.5 mg/kg), and glycopyrrolate (0.01 mg/kg). Endotracheal intubation was performed, and a surgical plane of anesthesia was maintained with 100% oxygen and isoflurane (1%–3%). An introducer sheath was surgically inserted in the jugular vein for administration of IV fluids and blood collection. For those cohorts where the study drug was administered IV, a second introducer sheath was placed in the contralateral jugular vein. An enema (100–150 mL) was administered under anesthesia, followed by irrigation of the rectum.

### 2.5 Rectal hyperthermia and temperature monitoring

Two T-type thermocouples (accuracy: ±0.5 °C) were affixed to the heating tube at 6.5 cm and 12.5 cm positions using silicon tape and connected to a LogMaster 4-Channel Logger (ThermoWorks, Utah, United States), which measured temperature every second (Figure 1C). For analysis, a rolling average of the current and prior 9 measurements was used. The assembled rectal heating device was equilibrated to 44 °C by recirculating warm water prior to insertion into the rectum. Hyperthermia was maintained for 1 h from the start of drug infusion. For the IV LTLD cohort where the rectum was not heated, the rectal heating device was inserted but maintained at 37 °C.

### 2.6 Arterial access and imaging

For IA drug delivery, introducer sheaths were surgically placed in the carotid arteries bilaterally. Catheters were advanced through the sheaths and diagnostic pelvic angiography was performed and the arteries supplying the rectum were visualized. Then, catheters were advanced into the internal iliac arteries bilaterally. The catheter tips were positioned in the distal internal iliac arteries at the level of the caudal aspect of the obturator foramina in the posterior-anterior imaging projection.

### 2.7 Treatments

The swine were divided into four treatment groups (n = 2/group): (1) IV LTLD (Thermodox®, Celsion/Immunon, New Jersey, United States), (2) IA doxorubicin with hyperthermia, (3) IV LTLD with hyperthermia, and (4) IA LTLD with hyperthermia. Drug formulations were infused over 30-min at a dose of 0.7 mg/kg. For IA drug delivery, the drug dose was divided equally between the two catheters in the internal iliac arteries. Catheters were connected to an automatic dual syringe injector to deliver the entire dose in 30 min. Drug infusion began following temperature equilibration at 44 °C inside the rectum.

### 2.8 Pharmacokinetics analysis

Venous blood samples (3 mL) were collected at baseline and sequentially for 1 h from the start of infusion. Plasma was separated by centrifugation, and doxorubicin was quantified by LC/MS. A non-compartmental analysis was employed to determine pharmacokinetic parameters. Maximum plasma concentration (C_max_) and time to C_max_ (T_max_) were observed values. The elimination rate constant and terminal half-life were calculated from the log-linear regression of terminal exponential phase data points. The area under the concentration-time curve (AUC_0–60min_) was calculated from the beginning of the drug infusion to the time of the last measurable plasma concentration using the trapezoid method and extrapolating to infinity (AUC_0-_  ∞ ) by dividing the last measurable plasma concentration by the terminal exponential rate constant. Clearance (CL) was calculated as dose/AUC_0-_  ∞ .

### 2.9 Tissue distribution and quantification of doxorubicin

One hour after the start of drug infusion, swine were euthanized under general anesthesia by administration of Beuthanasia-D or equivalent (Pentobarbital Sodium 390 mg/mL and Phenytoin Sodium 50 mg/mL; 1 mL/10 lbs IV), consistent with current AVMA guidelines for euthanasia. Immediately following euthanasia, the rectum was exposed via a midline abdominal incision, marked at cranial and caudal aspects using sutures, and dissected *en bloc*. The rectum was opened with a midline incision and divided into 12–16 pieces, approximately 2 × 2 cm. For each animal, the mean concentration of doxorubicin in eight pieces taken from ventral, dorsal, cranial, and caudal aspects was determined. Samples of perirectal fat and the heart were also acquired. Tissues were rapidly frozen in liquid nitrogen. Frozen tissues were mounted with Tissue-Tek cryoadhesive (Sakura Finetek, Torrance, CA) and cut into 10 μm cross-sections for fluorescence imaging. Doxorubicin was extracted from homogenized rectum, heart, and perirectal fat samples and quantified by LC/MS.

### 2.10 Qualitative analysis of drug distribution

Doxorubicin distribution in the rectal wall was assessed by fluorescence microscopy (Olympus VS200-6 FL, Center Valley, Pennsylvania, United States; excitation 468 nm, emission 635 nm) of transverse rectal wall sections. Images of intrinsic doxorubicin fluorescence were pseudo-colored yellow. All images were captured with identical exposure and window/level settings.

### 2.11 Statistical analysis

Comparisons of mean tissue doxorubicin concentrations and pharmacokinetic parameters between groups were performed using one-way ANOVA with a single pooled variance (GraphPad Prism v9.0.0). Post hoc analyses were performed using Tukey’s multiple comparison test. T_max_ is presented as median and range.

## 3 Results

### 3.1 Qualitative characterization of rectal heating in a thermochromic phantom

**FIGURE 2:**
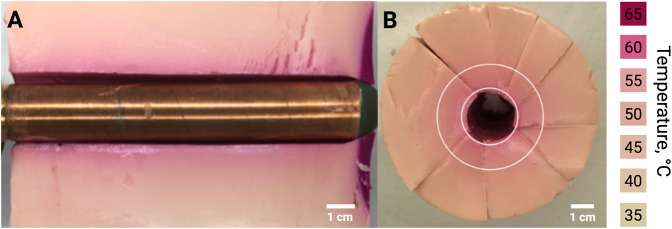
Spatial uniformity of heating in a thermochromic phantom. **(A)** Longitudinal section of thermochromic phantom containing the rectal heating device after heating for 1 h with 65 °C water; **(B)** Cross section of thermochromic phantom with rectal heating device removed. Color change from beige to pink and visible along the tube in **(A)** and between the concentric rings in **(B)**, indicates maximum temperature reached (Mikhail et al., 2016).

After 1 hour of circulating 65 °C water through the rectal heating device embedded in the thermochromic phantom, a uniform color change was observed along both the length and circumference of the device (Figure 2). This temperature was selected to ensure visible color change in the phantom.

### 3.2 Computational modeling of tissue heating and drug delivery

**FIGURE 3:**
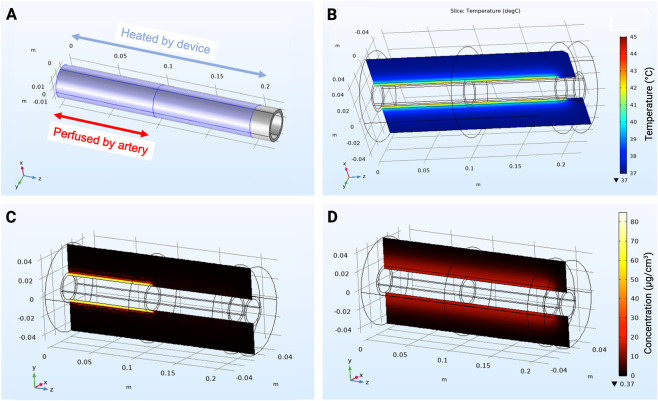
Computational modeling. **(A)** Model geometry and parameters. For simulation of IA and IV delivery of LTLD, the full length of the model rectum was heated. However, for simulation of IA LTLD delivery, only half the length of the model rectum was perfused by an artery selectively catheterized for drug delivery. **(B)** Estimated tissue temperature following 60 min of heating. Estimated DOX concentration in the rectal wall following 30-min **(C)** intra-arterial and **(D)** intravenous infusion of LTLD and 60-min of heating.

Computational simulations were performed to estimate tissue temperature and doxorubicin distribution in the rectal wall (Figure 3). The model geometry reflected the experimental setup with full-length heating for IV and IA LTLD simulations. For simulation of IA drug infusion, perfusion was included in only half of the model to mimic selective arterial catheterization. After 60 min of heating, tissue temperatures were predicted to reach the threshold for doxorubicin release from liposomes up to a few millimeters away from the rectal lumen. Simulations also suggested doxorubicin accumulation in the heated rectum following both IA and IV LTLD infusion, with differing spatial distribution patterns. The former appeared only in regions perfused by catheterized arteries and was more concentrated near the lumen, whereas the latter appeared at more moderate concentrations but with greater transverse and longitudinal distribution.

### 3.3 Arterial access and imaging

**FIGURE 4:**
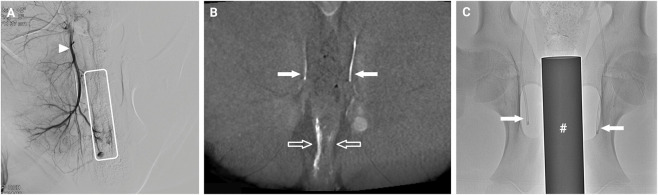
Angiography and catheter placement. **(A)** Angiography of the distal right internal iliac artery (arrowhead). Contrast enhancement of the distal rectum is indicated within the outlined box. **(B)** Coronal reconstruction of a cone-beam CT angiogram shows bilateral catheters (filled arrows) positioned in the internal iliac arteries and enhancement of the caudal portion of the rectum (unfilled arrows). **(C)** Anterior-posterior X-ray image of rectal heating device (crosshatch) inside the rectum of a swine, including the arterial catheters.

Bilateral catheter placement in the internal iliac arteries was confirmed by angiography (Figure 4A). Catheter localization and rectal perfusion were confirmed using contrast-enhanced cone-beam CT (Figure 4B). The position of the rectal heating device within the rectum and the catheters was confirmed by x-ray (Figure 4C).

### 3.4 *In vivo* temperature measurements

**FIGURE 5:**
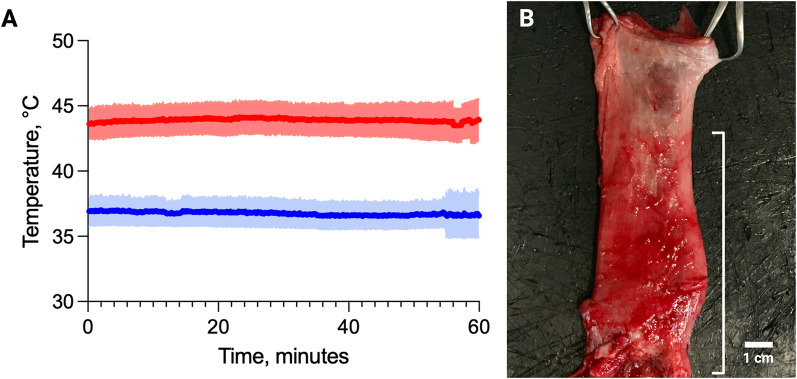
**(A)** Average temperature (±SD) at the interface of the rectal heating device and rectal lumen with (red) and without (blue) hyperthermia. **(B)** Explanted rectum from a swine treated with IA LTLD displaying pronounced red discoloration of the distal rectum and anus (bracket), corresponding to the accumulation of doxorubicin, which is red in color, in tissue exposed to localized heating by the rectal heating device.

During hyperthermia, the two thermocouples attached to the proximal and distal ends of the rectal heating device recorded mean temperatures of 43.5 °C and 44.3 °C, respectively (Figure 5A), indicating maintenance of the target hyperthermic range throughout the procedure.

### 3.5 Procedural outcomes and gross pathology

All animals tolerated the procedures without adverse events. No macroscopic signs of thermal injury to the rectum were observed. In animals receiving LTLD and hyperthermia, a sharply demarcated red discoloration was evident in the lower rectum and anus, most profoundly following IA LTLD delivery, corresponding to the region supplied by the catheterized vessels and exposed to the rectal heating device. This discoloration was consistent with doxorubicin accumulation (Figure 5B).

### 3.6 Fluorescence imaging of doxorubicin distribution

**FIGURE 6:**
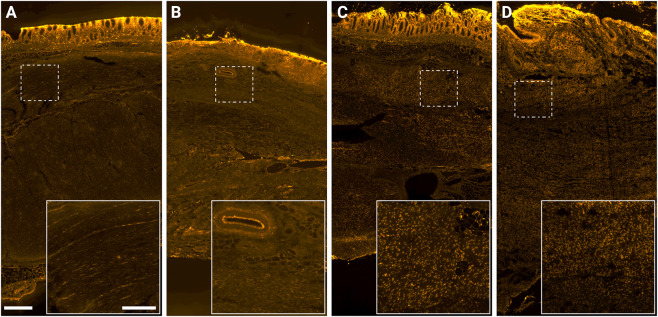
Doxorubicin fluorescence (pseudo-colored yellow) in transverse rectal wall sections from swine that received: **(A)** IV LTLD; **(B)** IA doxorubicin + HT; **(C)** IV LTLD + HT; and **(D)** IA LTLD + HT. The scale bar represents 500 µm for all main images and 200 µm for insets.

Fluorescence imaging of rectal wall cross sections revealed intrinsic doxorubicin fluorescence, with the most pronounced deposition observed in swine that received IV or IA LTLD combined with hyperthermia. In these treatment groups, doxorubicin was distributed throughout the rectal wall including the muscularis (Figure 6). The punctate fluorescence pattern, especially evident in LTLD-treated swine in combination with hyperthermia, is consistent with nuclear uptake of doxorubicin.

### 3.7 Pharmacokinetic analysis

**FIGURE 7:**
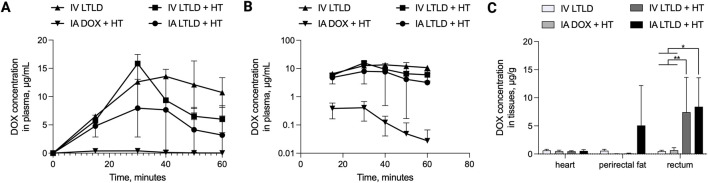
Plasma pharmacokinetics and biodistribution. **(A)** Mean plasma doxorubicin concentration measured from the beginning of drug infusion (0.7 mg/kg). **(B)** Semi-logarithmic plot of plasma doxorubicin concentrations. **(C)** Mean doxorubicin concentration in the heart, perirectal fat, and rectum 60 min after the start of drug infusion. For within-tissue type comparisons, *p < 0.1, **p < 0.01.

Plasma doxorubicin concentrations during treatment are shown in Figure 7A, and the corresponding pharmacokinetic parameters are summarized in Table 1. A complete list of p-values for comparisons between treatment groups is provided in the Supplementary Material. Compared to IA doxorubicin + HT, the values of AUC_0–60min_ and C_max_ were greater, and clearance (CL) was lower for IV LTLD, IV LTLD + HT, and IA LTLD + HT. The elimination half-life (t_1/2 elimination_) was longest for IV LTLD compared to IA doxorubicin + HT, IV LTLD + HT, and IA LTLD + HT.

**TABLE 1:** Pharmacokinetic parameters for non-compartmental analysis of doxorubicin in plasma.

Parameter	IV LTLD	IA doxorubicin + HT	IV LTLD + HT	IA LTLD + HT
AUC_0–60min_ (µg/mL·min)	544 ± 148	12.7 ± 8.36	424 ± 85.6	305 ± 221
AUC_0-_ ∞ (µg/mL·min)	3,656 ± 494.5	13.9 ± 8.04	962.1 ± 695.5	380 ± 308
C_max_ (µg/mL)	12.6 ± 4.85	0.410 ± 0.260	12.8 ± 4.34	8.54 ± 5.92
T_max_ (min)	30 (0)	22.5 (15–30)	35 (30–40)	35 (30–40)
CL (mL/min)	13.9 ± 2.16	3,959 ± 2006	68.4 ± 48.5	175 ± 130
t_1/2 elimination_ (min)	205 ± 27.8	13.2 ± 2.23	52.2 ± 49.6	14.3 ± 3.60

Data are mean ± SD except T_max_ (median, range). P-values are provided in Supplementary Table S1 (Supplementary Material). AUC_0-_  ∞ : Area Under the Curve from zero to infinity; AUC_0–60min_: Area Under the Curve from zero to 60 minutes, the last timepoint; CL: clearance; C_max_: maximum doxorubicin plasma concentration; T_max_: time to C_max_; t_1/2 elimination_: elimination half-life.

Doxorubicin concentrations in the rectal wall were highest in swine that received either IV LTLD + HT or IA LTLD + HT compared to IV LTLD (p = 0.0651 and 0.0405, respectively) or IA doxorubicin + HT (p = 0.0707 and 0.0438, respectively) (Figure 7B). Mean doxorubicin concentrations in full thickness samples of the rectal wall were 0.49 ± 0.16 (IV LTLD); 0.67 ± 0.46 (IA doxorubicin + HT); 7.45 ± 6.18 (IV LTLD + HT); and 8.41 ± 5.15 μg/g (IA LTLD + HT) (Figure 7C). Low doxorubicin levels were detected in the heart across all groups (Figure 7C). One swine treated with IA LTLD + HT had high doxorubicin concentration in the perirectal fat.

## 4 Discussion

Direct IA infusion of chemotherapy is a well-established clinical practice for liver-directed therapy of hepatocellular carcinoma and colorectal cancer liver metastases, but has not been explored for primary rectal cancer. Local drug delivery can reduce systemic exposure and increase drug concentrations in targeted tumors. Localized mild hyperthermia is an effective strategy for enhancing targeted drug delivery following IV administration of LTLD, but the pharmacokinetics and tissue deposition of doxorubicin after direct IA administration of LTLD to heated organs have not been previously characterized. In this study, we compared plasma pharmacokinetics and doxorubicin concentrations in the rectum of swine following IA doxorubicin, IV LTLD, and IA LTLD, using a custom device to heat the rectum, and IV LTLD without heating. IA and IV LTLD infusions resulted in similar doxorubicin concentrations in the rectum and achieved significantly higher tissue levels than IA free doxorubicin, when combined with local hyperthermia and IV LTLD under normothermic conditions, as measured 30 min after completion of drug infusion.

This finding suggests that temperature-triggered intravascular drug release, rather than the arterial route of administration alone, is the primary determinant of local tissue deposition. Notably, IV LTLD and IA LTLD combined with hyperthermia exhibited similar plasma AUC values over the 0–60 min experimental timeframe, suggesting that systemic pharmacokinetics during the heating period play a central role in determining local drug delivery. Prior studies of thermosensitive liposomal doxorubicin have shown that local tissue uptake is maximized when circulating blood concentrations of doxorubicin are elevated during hyperthermia and that systemic plasma exposure during the heating period correlates with drug accumulation in heated tissues (Rossmann et al., 2017; Sebeke et al., 2022). In this context, the relatively long circulation time of LTLD may diminish the incremental benefit of regional arterial delivery compared with intravenous administration, provided that adequate systemic exposure is sustained during localized hyperthermia.

The substantial increase in doxorubicin deposition in the rectum for IV or IA LTLD combined with hyperthermia compared to IV LTLD alone is consistent with the concept that local hyperthermia triggers rapid doxorubicin release from liposomes, enabling efficient tissue uptake (Haemmerich et al., 2023; Manzoor et al., 2012; Li et al., 2010; Ten Hagen et al., 2021). Notably, despite the immediate availability of free doxorubicin for extravasation after IA administration, both IA and IV LTLD provided superior tissue delivery when combined with hyperthermia, likely due to prolonged plasma residence time of encapsulated doxorubicin—reflected by higher AUC values for the liposomal formulations—that resulted in sustained release during the heating period. Despite the higher AUC of LTLD formulations compared to free doxorubicin, doxorubicin concentrations in the heart were not elevated reflecting entrapment of the drug in the liposomes during circulation enabling targeted delivery to the heated rectum.

Effective LTLD-mediated drug delivery requires precise heating of the target tissue above the temperature threshold for liposomal drug release, without causing thermal injury to tissues or blood vessels. Our team has previously demonstrated the ability to deliver doxorubicin to the wall of the urinary bladder via IV infusion of LTLD in combination with bladder-localized mild hyperthermia by intravesical warm water recirculation (Mikhail et al., 2017). However, there are currently no commercial medical devices capable of inducing mild hyperthermia in a temperature range adequate to trigger drug release from LTLD and localized to the rectum. A prototype rectal heating device was developed capable of providing uniform conductive heating at 44 °C along its length and circumference. This method of heating was sufficient to trigger the release of doxorubicin from the liposomes, as demonstrated by the tenfold increase in doxorubicin concentration in the rectal wall following delivery of IV LTLD when combined with hyperthermia of the rectum compared to normothermic conditions. Doxorubicin fluorescence was detected in the mucosal and muscle layers of the rectal wall, including extravascular compartments, indicating sufficient heat transfer for drug release following IV and IA delivery of LTLD. In one swine, elevated doxorubicin concentration was observed in perirectal fat following IA LTLD combined with hyperthermia. The significance of this isolated finding requires further exploration. In addition, although speculative, this device may be used to augment other therapies for which hyperthermia is beneficial, such as radiation therapy.

Computational modeling indicated that the spatial distribution of doxorubicin in the rectal wall may differ between IV and IA LTLD delivery, with IA delivery potentially allowing more targeted deposition via selective catheterization. However, we did not observe significant differences in overall drug deposition between these two routes of administration when combined with hyperthermia, albeit without quantitative assessment of doxorubicin concentrations in different layers of the rectal wall. Infusion of LTLD into the distal internal iliac arteries, as performed in this study, may result in higher local intravascular LTLD concentrations than IV administration; however, much of the infused drug traverses non-heated tissues supplied by the internal iliac arteries and is subsequently recirculated. More selective arterial delivery could increase local intravascular LTLD concentrations; however, any potential benefit would likely depend on sufficiently rapid intravascular drug release during the first pass through adequately heated tissue, prior to dilution upon systemic recirculation.

This study has several limitations. Healthy swine were used due to their anatomic similarity to humans, enabling the use of clinical-scale intra-arterial catheters and devices; however, heating, drug transport, and drug clearance may differ in tumor-bearing rectal tissue. Small group sizes and inter-subject variability limited statistical inference; however, consistent trends in pharmacokinetic and tissue accumulation measures support the mechanistic role of hyperthermia-triggered intravascular drug release. Doxorubicin, while not standard in rectal cancer therapy, was used as a model compound due to its intrinsic fluorescence and established thermosensitive liposomal formulation. Standard rectal cancer drugs such as 5-fluorouracil and oxaliplatin differ in physicochemical properties, which may affect their suitability for thermosensitive liposomal encapsulation; however, the delivery platform demonstrated here may be extendable to other therapeutics as appropriate formulations are developed. Plasma pharmacokinetics were assessed over a 60-min period, which may have limited detection of longer-term differences between groups, and doxorubicin measurements did not distinguish between free and liposome-encapsulated drug. Although rectal tissue concentrations were measured at a single time point in this dynamic process, higher systemic exposure was accompanied by higher rectal tissue concentrations, providing insight into the kinetics of hyperthermia-triggered drug delivery. Finally, while general anesthesia was required for the conduct of the study in an animal model, transarterial therapies and endoluminal procedures in clinical practice are commonly performed under conscious sedation or without sedation. Thermal tissue effects and tolerance in humans may differ, and future clinical translation will require optimization of heating parameters and incorporation of automated safety features, including real-time temperature monitoring and rapid shutoff controls, to maintain an appropriate safety margin and facilitate delivery under minimal or no sedation.

Despite advances in neoadjuvant chemoradiotherapy for rectal cancer, only a subset of patients achieve pathologic complete response, highlighting the need for alternative therapeutic strategies that may enable organ preservation. The integration of thermosensitive liposomal drug delivery with localized hyperthermia represents a mechanism-based, site-specific approach for spatially controlled chemotherapy delivery and provides a platform for further investigation in disease-relevant settings.

## 5 Conclusion

These exploratory findings support the feasibility of combining thermosensitive liposomal drug delivery, localized rectal hyperthermia, and intra-arterial catheter-based delivery as a platform for site-specific drug targeting. More selective catheterization of tissue- or tumor-feeding arteries could be evaluated in future studies to determine whether increased first-pass exposure during localized hyperthermia influences local drug delivery.

## Data Availability

The original contributions presented in the study are included in the article/Supplementary Material. Further inquiries can be directed to the corresponding author.

## References

[B2] Benson A. B. Venook A. P. Al-Hawary M. M. Cederquist L. Chen Y. J. Ciombor K. K. (2018). Rectal cancer, version 2.2018, NCCN Clinical practice guidelines in oncology. J. Natl. Compr. Canc Netw. 16, 874–901. 10.6004/jnccn.2018.0061 30006429 PMC10203817

[B3] Borys N. Dewhirst M. W. (2021). Drug development of lyso-thermosensitive liposomal doxorubicin: combining hyperthermia and thermosensitive drug delivery. Adv. Drug Deliv. Rev. 178, 113985. 10.1016/j.addr.2021.113985 34555486

[B4] Burke C. Dreher M. R. Negussie A. H. Mikhail A. S. Yarmolenko P. Patel A. (2018). Drug release kinetics of temperature sensitive liposomes measured at high-temporal resolution with a millifluidic device. Int. J. Hyperth. 34, 786–794. 10.1080/02656736.2017.1412504 29284329 PMC6145460

[B5] Collette L. Bosset J.-F. Dulk M. D. Nguyen F. Mineur L. Maingon P. (2007). Patients with curative resection of cT3-4 rectal cancer after preoperative radiotherapy or radiochemotherapy: does anybody benefit from adjuvant fluorouracil-based chemotherapy? A trial of the european organisation for Research and treatment of Cancer Radiation Oncology Group. J. Clin. Oncol. 25, 4379–4386. 10.1200/JCO.2007.11.9685 17906203

[B6] Eranki A. Mikhail A. S. Negussie A. H. Katti P. S. Wood B. J. Partanen A. (2019). Tissue-mimicking thermochromic phantom for characterization of HIFU devices and applications. Int. J. Hyperth. 36, 518–529. 10.1080/02656736.2019.1605458 31046513 PMC6625350

[B7] Fokas E. Liersch T. Fietkau R. Hohenberger W. Beissbarth T. Hess C. (2014). Tumor regression grading after preoperative chemoradiotherapy for locally advanced rectal carcinoma revisited: updated results of the CAO/ARO/AIO-94 trial. J. Clin. Oncol. 32, 1554–1562. 10.1200/JCO.2013.54.3769 24752056

[B8] Gasselhuber A. Dreher M. R. Partanen A. Yarmolenko P. S. Woods D. Wood B. J. (2012). Targeted drug delivery by high intensity focused ultrasound mediated hyperthermia combined with temperature-sensitive liposomes: computational modelling and preliminary *in vivo* validation. Int. J. Hyperth. 28, 337–348. 10.3109/02656736.2012.677930 22621735 PMC7641876

[B9] Gérard J. P. Conroy T. Bonnetain F. Bouché O. Chapet O. Closon-Dejardin M. T. (2006). Preoperative radiotherapy with or without concurrent fluorouracil and leucovorin in T3-4 rectal cancers: results of FFCD 9203. J. Clin. Oncol. 24, 4620–4625. 10.1200/JCO.2006.06.7629 17008704

[B10] Haemmerich D. Ramajayam K. K. Newton D. A. (2023). Review of the delivery kinetics of thermosensitive liposomes. Cancers 15, 398. 10.3390/cancers15020398 36672347 PMC9856714

[B11] Li L. Ten Hagen T. L. M. Schipper D. Wijnberg T. M. Van Rhoon G. C. Eggermont A. M. M. (2010). Triggered content release from optimized stealth thermosensitive liposomes using mild hyperthermia. J. Control Release 143, 274–279. 10.1016/j.jconrel.2010.01.006 20074595

[B12] Lim Y. J. Kim Y. Kong M. (2019). Adjuvant chemotherapy in rectal cancer patients who achieved a pathological complete response after preoperative chemoradiotherapy: a systematic review and meta-analysis. Sci. Rep. 9, 10008. 10.1038/s41598-019-46457-5 31292517 PMC6620266

[B13] Manzoor A. A. Lindner L. H. Landon C. D. Park J.-Y. Simnick A. J. Dreher M. R. (2012). Overcoming limitations in nanoparticle drug delivery: triggered, intravascular release to improve drug penetration into tumors. Cancer Res. 72, 5566–5575. 10.1158/0008-5472.CAN-12-1683 22952218 PMC3517817

[B14] Mikhail A. S. Negussie A. H. Graham C. Mathew M. Wood B. J. Partanen A. (2016). Evaluation of a tissue-mimicking thermochromic phantom for radiofrequency ablation. Med. Phys. 43, 4304–4311. 10.1118/1.4953394 27370145 PMC4912564

[B15] Mikhail A. S. Negussie A. H. Pritchard W. F. Haemmerich D. Woods D. Bakhutashvili I. (2017). Lyso-thermosensitive liposomal doxorubicin for treatment of bladder cancer. Int. J. Hyperth. 33, 733–740. 10.1080/02656736.2017.1315459 28540814 PMC7676871

[B16] Needham D. Anyarambhatla G. Kong G. Dewhirst M. W. (2000). A new temperature-sensitive liposome for use with mild hyperthermia: characterization and testing in a human tumor xenograft model. Cancer Res. 60, 1197–1201. 10728674

[B17] Negussie A. H. Partanen A. Mikhail A. S. Xu S. Abi-Jaoudeh N. Maruvada S. (2016). Thermochromic tissue-mimicking phantom for optimisation of thermal tumour ablation. Int. J. Hyperth. 32, 1–5. 10.3109/02656736.2016.1145745 27099078 PMC7831156

[B18] Rahbari N. N. Elbers H. Askoxylakis V. Motschall E. Bork U. Büchler M. W. (2013). Neoadjuvant radiotherapy for rectal cancer: meta-analysis of randomized controlled trials. Ann. Surg. Oncol. 20, 4169–4182. 10.1245/s10434-013-3198-9 24002536

[B19] Rossmann C. McCrackin M. A. Armeson K. E. Haemmerich D. (2017). Temperature sensitive liposomes combined with thermal ablation: effects of duration and timing of heating in mathematical models and *in vivo* . PLoS One 12, e0179131. 10.1371/journal.pone.0179131 28604815 PMC5467840

[B20] Sauer R. Becker H. Hohenberger W. Rödel C. Wittekind C. Fietkau R. (2004). Preoperative versus postoperative chemoradiotherapy for rectal cancer. N. Engl. J. Med. 351, 1731–1740. 10.1056/NEJMoa040694 15496622

[B21] Sebeke L. C. Castillo Gómez J. D. Heijman E. Rademann P. Simon A. C. Ekdawi S. (2022). Hyperthermia-induced doxorubicin delivery from thermosensitive liposomes via MR-HIFU in a pig model. J. Control Release 343, 798–812. 10.1016/j.jconrel.2022.02.003 35134460

[B1] SEER*Explorer: an interactive website for SEER cancer statistics. SEER*Explorer: an interactive website for SEER cancer statistics. (2025). Bethesda, Maryland: National Cancer Institute. Available online at: https://seer.cancer.gov/statistics-network/explorer/(Accessed June 25, 2025).

[B22] Siegel R. L. Fedewa S. A. Anderson W. F. Miller K. D. Ma J. Rosenberg P. S. (2017). Colorectal cancer incidence patterns in the United States, 1974-2013. J. Natl. Cancer Inst. 109. 10.1093/jnci/djw322 28376186 PMC6059239

[B23] Siegel R. L. Giaquinto A. N. Jemal A. (2024). Cancer statistics, 2024. CA A Cancer Journal Clinicians 74, 12–49. 10.3322/caac.21820 38230766

[B24] Tak W. Y. Lin S. M. Wang Y. Zheng J. Vecchione A. Park S. Y. (2018). Phase III HEAT study adding lyso-thermosensitive liposomal doxorubicin to radiofrequency ablation in patients with unresectable hepatocellular carcinoma lesions. Clin. Cancer Res. 24, 73–83. 10.1158/1078-0432.CCR-16-2433 29018051

[B25] Ten Hagen T. L. M. Dreher M. R. Zalba S. Seynhaeve A. L. B. Amin M. Li L. (2021). Drug transport kinetics of intravascular triggered drug delivery systems. Commun. Biol. 4, 920. 10.1038/s42003-021-02428-z 34321602 PMC8319190

[B26] Thompson H. M. Omer D. M. Lin S. Kim J. K. Yuval J. B. Verheij F. S. (2024). Organ preservation and survival by clinical response grade in patients with rectal cancer treated with total neoadjuvant therapy: a secondary analysis of the OPRA randomized clinical trial. JAMA Netw. Open 7, e2350903–e. 10.1001/jamanetworkopen.2023.50903 38194231 PMC10777257

[B27] van Valenberg F. J. P. Brummelhuis I. S. G. Lindner L. H. Kuhnle F. Wedmann B. Schweizer P. (2021). DPPG(2)-based thermosensitive liposomes with encapsulated doxorubicin combined with hyperthermia lead to higher doxorubicin concentrations in the bladder compared to conventional application in pigs: a rationale for the treatment of muscle-invasive bladder cancer. Int. J. Nanomedicine 16, 75–88. 10.2147/IJN.S280034 33447028 PMC7802347

[B28] Verkuijl S. J. Jonker J. E. Trzpis M. Burgerhof J. G. M. Broens P. M. A. Furnée E. J. B. (2021). Functional outcomes of surgery for colon cancer: a systematic review and meta-analysis. Eur. J. Surg. Oncol. 47, 960–969. 10.1016/j.ejso.2020.11.136 33277056

[B29] Wood B. J. Poon R. T.-P. Locklin J. K. Dreher M. R. Ng K. K. Eugeni M. (2012). Phase I study of heat-deployed liposomal doxorubicin during radiofrequency ablation for hepatic malignancies. J. Vasc. Interv. Radiol. 23, 248–255. 10.1016/j.jvir.2011.10.018 22178041 PMC3264789

[B30] Zagar T. M. Vujaskovic Z. Formenti S. Rugo H. Muggia F. O'Connor B. (2014). Two phase I dose-escalation/pharmacokinetics studies of low temperature liposomal doxorubicin (LTLD) and mild local hyperthermia in heavily pretreated patients with local regionally recurrent breast cancer. Int. J. Hyperth. 30, 285–294. 10.3109/02656736.2014.936049 25144817 PMC4162656

