# Supplementary material for: Hyperthermia-targeted rectal delivery of thermosensitive liposomal doxorubicin via intra-arterial and intravenous administration
Source: Front Pharmacol. 2026 Feb 18;17:1760944. doi: 10.3389/fphar.2026.1760944 (PMC12957794; doi:10.3389/fphar.2026.1760944)
Supplement: Supplementary file 1 [file Table1.docx]

**Supplementary Material**

Supplementary Table 1. Pharmacokinetic parameter comparison p-values adjusted for multiple comparisons.

| **AUC_0-last_** | IA DOX+HT | IV LTLD+HT | IA LTLD+HT |
| --- | --- | --- | --- |
| IV LTLD | 0.0191 | 0.4396 | 0.163 |
| IA DOX+HT |  | 0.0423 | 0.1044 |
| IV LTLD+HT |  |  | 0.4434 |
| **AUC_0-∞_** | IA DOX+HT | IV LTLD+HT | IA LTLD+HT |
| IV LTLD | 0.0129 | 0.0138 | 0.0067 |
| IA DOX+HT |  | 0.295 | 0.8487 |
| IV LTLD+HT |  |  | 0.6164 |
| **C_max_** | IA DOX+HT | IV LTLD+HT | IA LTLD+HT |
| IV LTLD | 0.1535 | >0.9999 | 0.794 |
| IA DOX+HT |  | 0.1484 | 0.3731 |
| IV LTLD+HT |  |  | 0.7766 |
| **CL** | IA DOX+HT | IV LTLD+HT | IA LTLD+HT |
| IV LTLD | 0.0562 | >0.9999 | 0.9983 |
| IA DOX+HT |  | 0.0588 | 0.0641 |
| IV LTLD+HT |  |  | 0.9995 |
| **t_1/2elimination_** | IA DOX+HT | IV LTLD+HT | IA LTLD+HT |
| IV LTLD | 0.0089 | 0.0201 | 0.009 |
| IA DOX+HT |  | 0.5758 | >0.9999 |
| IV LTLD+HT |  |  | 0.5937 |

AUC_0-∞_: Area Under the Curve from zero to infinity

AUC_0-last_: Area Under the Curve from zero to the last measurable timepoint

C_max_: Maximum plasma concentration

CL: Clearance

t_1/2elimination_: Elimination half-life
